# Oral Health Status, Behavior, and Knowledge of Patients with Cardiovascular Disease and Associated Risk Factors in Odisha: A Cross-Sectional Survey

**DOI:** 10.3390/dj13090401

**Published:** 2025-09-01

**Authors:** Lora Mishra, Muskan Sharma, Naomi Ranjan Singh, Gathani Dash, Satya Ranjan Misra, Krzysztof Sokolowski, Manoj Kumar, Rupsa Das, Suresh Kumar Behera, Barbara Lapinska

**Affiliations:** 1 Department of Conservative Dentistry and Endodontics, Institute of Dental Sciences, Siksha ‘O’ Anusandhan, Bhubaneswar 751003, Odisha, India; ugpublicationids@gmail.com (M.S.); naomiranjansingh@soa.ac.in (N.R.S.); 2 Dental Department, Office of Superintendent, Community Health Centre, Sakhigopal, Puri 752014, Odisha, India; gathanidash@gmail.com; 3 Department of Oral Medicine and Radiology, Institute of Dental Sciences, Siksha ‘O’ Anusandhan, Bhubaneswar 751003, Odisha, India; satyamisra@soa.ac.in (S.R.M.); rupsadas@soa.ac.in (R.D.); 4 Department of Conservative Dentistry, Medical University of Lodz, 251 Pomorska St., 92-213 Lodz, Poland; krzysztof.sokolowski@umed.lodz.pl; 5 Department of Periodontics, Institute of Dental Sciences, Siksha ‘O’ Anusandhan, Bhubaneswar 751003, Odisha, India; manojkumar@soa.ac.in; 6 Department of Cardiology, Institute of Medical Sciences and SUM Hospital, Siksha ‘O’ Anusandhan, Bhubaneswar 751003, Odisha, India; sureshbehera@soa.ac.in; 7 Department of General Dentistry, Medical University of Lodz, 251 Pomorska St., 92-213 Lodz, Poland

**Keywords:** oral health, cardiovascular disease, self-reported oral health status, cross-sectional survey, public health

## Abstract

**Background:** Cardiovascular diseases (CVDs) are linked with poor oral health outcomes, yet data on oral health status, behaviors, and awareness among CVD patients in Odisha remain scarce. This study aimed to assess the self-reported oral health status, behaviors, and knowledge among patients with CVD and associated risk factors. **Methods:** A cross-sectional survey was conducted among 391 patients aged 40–80 years attending dental and cardiology OPDs at a tertiary care center in Bhubaneswar. Participants were grouped into control, at-risk, and established CVD categories. A 24-item questionnaire and panoramic radiographic examination were used to assess oral health. Data were analyzed using the chi-squared test and Kappa statistics. **Results:** Patients with established CVD reported significantly higher prevalence of oral health problems, poor oral hygiene behaviors, and lower awareness compared to controls. Clinical findings revealed higher rates of periodontal bone loss, caries, and periapical radiolucency in the CVD group. Agreement between radiographic examiners was high. **Conclusions:** There is a substantial burden of oral disease and poor oral health awareness among patients with CVD in Odisha. These findings emphasize the need for integrated oral health education and care protocols in cardiovascular patient management.

## 1. Introduction

The prevalence of systemic illnesses is on the rise among the Indian populace, with cardiovascular diseases emerging as a significant focal point of public health attention [1,2,3,4,5]. Recent demographic research has brought attention to the rising occurrence of systemic illnesses, with cardiovascular disease (CVD) at the forefront, along with metabolic and hematological disorders following closely [5,6]. This growing health burden emphasizes the need to comprehend its effects on different facets of well-being, including oral health.

Systemic illnesses, such as CVD, can frequently result in oral symptoms, adding complexity to dental care and treatment [7,8,9]. Despite the widespread occurrence of systemic diseases, patients often have insufficient knowledge about their health conditions and prescribed medications. This lack of awareness presents difficulties for dental professionals and raises the potential for complications during dental treatments due to possible interactions with medication [10,11,12].

Furthermore, the connection between systemic illnesses and oral health makes managing both conditions more complex [13,14,15]. Persistent conditions such as diabetes, hypertension, and cardiovascular diseases frequently manifest in oral indications that can exacerbate overall health concerns [16]. The extended inflammation linked to both systemic and oral ailments in older adults underscores the intricate connection between oral and systemic well-being [17]. Despite the integral role of oral health in overall well-being, a significant proportion of the Indian population primarily seeks dental treatment for symptomatic relief rather than preventive care [18]. This trend is particularly pronounced in rural areas, where limited access to oral health education perpetuates inadequate oral hygiene practices [19]. Addressing these disparities in oral health knowledge and behaviors is imperative to promote better oral health outcomes and overall health among individuals with cardiovascular disease.

Several studies emphasize the importance of self-reporting for understanding oral health in CVD patients and provide a more detailed picture than clinical assessments alone [20,21,22,23,24,25,26]. These findings suggest a bidirectional link, where not only can poor oral health potentially contribute to the onset or exacerbation of cardiovascular conditions, but CVD can also predispose patients to deteriorated oral health due to changes in lifestyle, medication side effects, or neglect of oral hygiene.

Patients’ perceptions are influenced by a myriad of factors, including cultural beliefs, socioeconomic status, and educational level, all of which play critical roles in the management of their oral and cardiovascular health [27,28,29,30,31,32,33,34,35,36].

However, previous studies reviewed point toward a gap in knowledge with respect to the Indian context, particularly in regions such as Odisha, where traditional healthcare practices, socio-economic variances, and cultural nuances add layers of complexity to the matrix of oral health and CVD. It is in this gap that this study is situated, offering a valuable contribution to a field that is clearly rich with potential for research and advancement in public health policies.

## 2. Aims and Objectives

### 2.1. Aim

The principal aim of this research was to evaluate self-reported oral health status and comprehend the dental hygiene behaviors and practices amongst patients diagnosed with cardiovascular disease in Odisha, India. Additional facets included assessing knowledge and awareness levels surrounding oral health within this demographic and discerning any disparities or gaps that may exist.

### 2.2. Objectives

This study aims to conduct a cross-sectional survey in Odisha to assess oral health among patients with cardiovascular disease (CVD) through self-reports. It will examine the prevalence of common oral health issues like dental caries, periodontal disease, and gingival bleeding, along with oral health behaviors such as dental visits, toothbrushing habits, and tobacco use. Additionally, this study will evaluate patient awareness of the oral health–CVD connection, compare findings with non-CVD individuals, and explore socio-demographic influences on oral health. Based on the insights gained, tailored recommendations will be developed to promote oral healthcare and improve overall health outcomes for CVD patients in Odisha.

## 3. Materials and Methods

### 3.1. Participant Selection

The cross-sectional survey (IEC Registration Number EC/NEW/INST/2022/3235) was administered to patients attending a dental college and a medical hospital of the university, from 25 October to 26 December 2023, for treatment or consultation, and who provided informed consent to participate in this study. To ensure a focus on the research aim and objectives, a purposive sampling technique was employed to recruit individuals diagnosed with CVD, with no severe medical conditions such as chronic illnesses (e.g., cancer, advanced diabetes), acute medical emergencies (e.g., stroke, severe infections, organ failure), or progressive disorders (e.g., neurodegenerative diseases) that typically demand ongoing medical treatment, hospitalization, or specialized care to manage symptoms and prevent complications.

Prior to inclusion in this study, all participants were provided with information outlining the purpose of the research, its significance, and what their participation would involve. Informed consent was obtained from each participant, emphasizing that their participation was voluntary and that they could withdraw at any time without any consequence to their care. Two examiners independently performed the radiographic assessments of oral health burden. Inter-examiner agreement was calculated using Kappa statistics, which showed high reliability.

### 3.2. Data Collection Procedure

Flyers were distributed in the waiting rooms of the medical institute, directing interested participants to the dental institute, where they received information about this study. Participants completed a self-administered questionnaire while waiting for their medical appointment. Participation was voluntary, and oral health information and dental services were provided regardless of participation. Written consent was obtained, and completion of the questionnaire took approximately 10 to 15 min. The Atherosclerotic Cardiovascular Disease (ASCVD) Risk assessment was performed using the ASCVD Risk Estimator Mobile Application 5.0 version developed by the American College of Cardiology.

### 3.3. Questionnaire Tool

The questionnaire, adapted from a validated instrument for assessing oral health status, behavior, and knowledge in pregnant women, was modified for the study population [26]. Items were guided by Andersen’s model to assess factors influencing access to dental care among people with CVD. It included items on oral health status and behavior of people with and without CVD and oral health knowledge. For our context, the tool underwent expert panel review for face and content validity, pilot testing among 20 CVD patients, and translation into Odia (local language), followed by back-translation into English to ensure cultural appropriateness. Knowledge of oral health was assessed based on a questionnaire with 12 questions related to oral health. The correct response was treated as a score of ‘0’ and the incorrect response as ‘1’. Then, the scores were added to find out the knowledge of oral health. The maximum score was 12 if one responded correctly to all 12 questions, and the minimum score was 0 if one responded incorrectly to all 12 questions. Thereafter, the descriptive statistics were calculated to assess the knowledge of oral health.

### 3.4. Sample Size

Sample size estimation was based on the previous research investigating the prevalence of endodontic diseases among CVD and cardiovascular disease risks (CVDR) patients [37]. In this study, the association of oral-health-related quality of life with endodontic factors in cardiovascular disease patient groups was mainly studied using the chi-squared test of independence. Therefore, the sample size determination was performed for the chi-squared test of independence using G*Power 3.1.9.4 software. Since the prevalence of endodontic factors has 2 categories and there are 3 groups, the degrees of freedom is 2.

Analysis: A priori: compute required sample size.

Input: Tail(s) = Two;

Effect size |ρ| = 0.18;

α err prob = 0.05;

Power (1-β err prob) = 0.95.

Output: Noncentrality parameter δ = 3.6183698;

Critical t = 1.9660811;

Df = 389;

Total sample size = 391;

Actual power = 0.9504731.

The sample size for this study is 514, which is higher than the minimum sample size of 391 to achieve a power of the test of 0.95 for a 0.05 level of significance.

### 3.5. Data Analysis

SPSS Version 24 was used for the analysis. Descriptive statistics were calculated for continuous variables (such as age, blood pressure, and cholesterol levels) as mean ± standard deviation (SD), median with interquartile range (IQR), and range (minimum to maximum). For categorical variables (such as gender, comorbidity status, and oral health behaviors), frequencies and percentages were reported. Inferential statistics were performed using chi-squared tests for categorical data to assess associations between variables, and independent sample t-tests or one-way ANOVA were applied for continuous variables to compare group differences. Pearson’s or Spearman’s correlation coefficients were used to assess relationships between continuous variables, depending on the normality of data distribution. Normality was checked using the Shapiro–Wilk test. All statistical analyses were conducted using SPSS Version 24, with a significance level set at *p* < 0.05. Potential confounding variables (age, sex, diabetes, hypertension, smoking, blood pressure, lipid levels, and medication use) were included in the multivariable logistic regression model, and possible effect modification was assessed by testing interaction terms (e.g., knowledge × diabetes, knowledge × hypertension).

This study is reported in accordance with the STROBE (Strengthening the Reporting of Observational Studies in Epidemiology) guidelines, and a completed checklist is provided as Supplementary Material.

## 4. Results

### 4.1. Demographic Status of Included Population

A sample of 514 patients with cardiovascular disease and associated risk factors was studied within the age group of 40–80 years. Table 1 presents distributions of patients by age, gender, status of comorbidity, habits, therapy, cardiovascular risks, and descriptive statistics of hemodynamic parameters. The sample cases were predominantly in the age group of 40–49 years (69.8%). The remaining were within the 50–80 years age group (30.2%). The mean age was 49.0 ± 7.9, and the median age with interquartile range (IQR) was 47 (43–52) years. Males were highly predominant in the sample with a share of 70.6%. Diabetes was the most prevalent comorbidity among the sample subjects, with 75.1% cases, followed by hypertension. About four-fifths had the habit of smoking (82.3%). About 8% patients were on statins therapy to lower their level of LDL or “bad” cholesterol that builds up in arteries. Similarly, 4.5% were on aspirin therapy to lower the risk of a heart attack. The mean systolic blood pressure (SBP) level was 130.3 ± 13.3 mmHg. The median (IQR) was 131.0 (119.8–140.0) mmHg. The range was (96.0–178.0). Low SBP is defined as lower than 120 mmHg; elevated SBP, 120 to 129 mmHg; stage I high blood pressure, 130–139 mmHg; stage II hypertension, 140 or higher; and hypertensive crisis,180 or more [38]. About a quarter of the sample cases had stage II hypertension—SBP of 140 or higher. Normal diastolic blood pressure (DBP) is defined as lower than 80 mmHg; stage I hypertension, 80 to 89 mmHg; stage II hypertension, 90 or more; and hypertensive crisis, 120 or more mmHg [38]. The mean DBP level was 82.7 ± 9.5 mmHg. The median (IQR) was 84.0 (77.0–89.0) mmHg. The range was 60.0–99.0. DBP also indicated that a quarter of the sample cases were stage II hypertension.

The mean total cholesterol (mg/dL) level was 174.4 ± 48.3 mg/dL. The median (IQR) was 166.9 (139.8–195.3) mg/dL. The range was (49.0–402.7) mg/dL. The mean HDL (mg/dL) level was 43.0 ± 7.4 mg/dL. The median (IQR) was 42.1 (39.8–45.2) mg/dL. The range was (11.9–84.0) mg/dL. The mean LDL (mg/dL) level was 104.5 ± 40.8 mg/dL. The median (IQR) was 98.2 (76.1–126.3) mg/dL. The range was (24.9–319.0) mg/dL. The TCL, HDL, and LDL analysis indicated that the majority of the patients had elevated cholesterol levels. Out of 514 patients, 53.3% had intermediate cardiovascular risk, while 14% and 16% had high and borderline cardiovascular risk, respectively.

### 4.2. Self-Reported Oral Health Status

The self-reported OHS was classified into five ordinal classes: Excellent, Very good, Good, Fair, and Poor. About one-fifth of patients classified their OHS as very good. The majority of them, i.e., 64.4% assessed OHS as good, and 15% assessed OHS as fair (Table 2).

As many as 43.2% reported one oral health problem, while 56.2% reported two or more oral health problems. Sample patients have reported several types of oral health problems; the most prevalent were sensitivity (161, 31.3%), followed by dry mouth (96, 18.7%) and teeth that do not look right (are broken, crooked, or discolored) (80, 15.6%). The other reported problems were cavities (68, 13.2%), bleeding/swollen/painful gums (56, 10.9%), toothache (26, 5.1%), and others (27, 5.3%). Nearly two-thirds of the sample patients (326, 63.4%) reported that dental problems affected what they eat.

Patients’ views on the importance of oral health problems with reference to overall health were obtained using a 10-point Likert scale, with scores 0–4 indicating low importance, 5 as neutral, and 6–10 as important to extremely important. As many as 153 (29.8%) patients adjudged oral health problems to be of low importance. Nearly half were neutral (278, 54.1%), i.e., making no differentiation between the importance of oral health and overall health. Nearly one-sixth rated oral health as important to extremely important (83, 16.1%) with respect to overall health.

### 4.3. Oral Health Behavior

The data on several aspects of oral health practices, such as the use of partial or full dentures, frequency of brushing teeth/dentures, oral hygiene products used, dental visits, and information about oral health care, are presented in Table 3.

About a quarter (24.5%) of respondents have used partial or full dentures. About one-fourth reported brushing their teeth a few times a week, and one-fifth reported brushing less than once a day. Little more than half (57.4%) reported brushing teeth once daily, and only 0.2% reported brushing teeth twice or more. Thus, the practice of brushing teeth emerged as an area of concern. Regarding the use of oral health products, the scenario is reasonably good with 37.4% using fluoride toothpaste, 33.9% using dental floss or other aids, and 28.6% using mouthwash. Nevertheless, there is a scope for further improvement.

Even though so many patients reported oral health problems, only 8.9% had visited a dentist during the last year. About 44.7% visited a dentist within the last one to two years, 35.2% during the last two to five years, and 7.8% beyond five years.

About 38.5% visited a private clinic, 44% visited a public clinic or hospital (government funded), and 12.8% visited other types of hospitals for oral health problems. When asked whether they had received information on oral health care since diagnosis, 44% responded affirmatively, and the remaining 56% negatively.

### 4.4. Association of Oral Health Status with Atherosclerosis Cardiovascular Disease (ASCVD) Risk Score

Oral health status was classified into three groups: Very good/Excellent, Good, and Fair (Table 4). As there was only one participant with an excellent OHS, this category was combined with the ‘Very Good’ group to form a single category labeled ‘Very Good/Excellent’ group. There was no significant association of OHS with ASCVD Risk Score (*p* = 0.561). This implied that the perception of oral health status does not make any significant difference to the ASCVD risk score.

### 4.5. Comparison of Knowledge Score with Oral Health Status

Patients with a very good/excellent perception of OHS had a mean knowledge score of 4.9 ± 1.4, with a median (IQR) score of 5 (3–6) (Table 5). Patients with a good perception of OHS had a mean knowledge score of 5.3 ± 1.1, with median (IQR) score of 5 (5–6), which was higher than very good/excellent group. Patients with fair perception of OHS had a mean knowledge score of 7.5 ± 2.0, with median (IQR) score of 9 (5–9), which was higher than very good/excellent group, which was higher than the mean and median knowledge score of patients with good oral health status. The difference in mean and median knowledge score was found to be significant (*p* ≤ 0.001). Knowledge score was moderately correlated with oral health status (Spearman’s ρ = −0.36, 95% CI: −0.42 to −0.29, *p* < 0.001).

### 4.6. Bivariate Associations (Chi-Squared/ANOVA, ASCVD vs. OHS, Knowledge vs. OHS)

On multivariable logistic regression, oral-health knowledge emerged as the only independent predictor of poor oral health status. Each one-point increase in the knowledge score was associated with a 34% higher odds of reporting poorer oral health (AOR 1.34, 95% CI 1.21–1.48, *p* < 0.001). Other factors, including age, sex, diabetes, hypertension, smoking, systolic and diastolic blood pressure, lipid measures, and medication use (statins, aspirin), were not statistically significant after adjustment. The model demonstrated acceptable performance (AUC 0.707) and calibration (Hosmer–Lemeshow χ^2^= 12.35, *p* = 0.133). A sensitivity analysis treating oral health status as an ordinal three-level outcome (very good/excellent, good, fair) produced consistent results for knowledge (OR 1.36, 95% CI 1.24–1.49, *p* < 0.001), reinforcing the robustness of this association.

## 5. Discussion

The survey findings from the dental college and medical hospital in Odisha provide crucial insights into oral health behaviors and knowledge among patients with cardiovascular disease and cardiovascular disease risk [37,39,40,41,42]. The data obtained reveal significant gaps in oral health maintenance, with a low percentage of participants engaging in routine dental visits. The infrequency of dental care among this patient population suggests an underlying problem of awareness and perception toward oral health’s role in cardiovascular well-being.

Inadequate oral-health knowledge often translates into suboptimal daily practices such as infrequent toothbrushing, poor interdental cleaning, and irregular preventive dental visits. These behaviors promote plaque accumulation and dysbiosis of the oral microbiota. Periodontal pathogens and their endotoxins can translocate into the systemic circulation, triggering low-grade inflammation characterized by increased C-reactive protein (CRP), interleukin-6 (IL-6), and tumor necrosis factor-α (TNF-α). Such inflammatory mediators contribute to endothelial dysfunction, insulin resistance, and atherogenesis—pathways central to cardiovascular risk. In addition, reduced abundance of nitrate-reducing commensals in dysbiotic oral biofilms may impair nitric oxide bioavailability, thereby influencing blood pressure regulation. Medications frequently used in cardiovascular disease (e.g., antihypertensives causing xerostomia) may further exacerbate microbial accumulation if patients lack knowledge to compensate with enhanced oral-hygiene behaviors. Oral pathogens have the potential to contribute to inflammation and atherosclerosis linked to CVD, requiring increased attention to dental health practices for these patients [43,44]. Timely dental visits are crucial not only for maintaining oral health but also for early detection of conditions influencing CVD progression. Therefore, optimizing oral health could be an important part of managing cardiovascular diseases.

Disparities in dental care visits and daily oral hygiene raise concerns about awareness levels and social determinants impacting health behaviors. Socioeconomic factors such as cost and access to care influence health-seeking behaviors. Additionally, psychological barriers and proactive preventive strategies within healthcare systems play a role in observed outcomes [45,46]. It is essential to address these complex challenges through targeted interventions aiming at improving overall oral health across diverse populations.

The level of knowledge among patients, as indicated by the survey, falls significantly short of what would be necessary to maintain good oral health and thereby mitigate potential cardiovascular consequences. Conducting a more comprehensive analysis of patient knowledge could help clarify whether these inadequacies stem from misunderstandings, lack of access to information, or ineffective communication from healthcare providers [46].

The evident disparities have profound implications not just for patients, but also for healthcare providers, who play a critical role in patient education and preventive care strategies [46]. The results underscore the necessity for healthcare providers to enhance patient education regarding the oral–systemic connection and encourage compliance with routine dental care. Healthcare providers also have a responsibility to identify systemic barriers that patients face and seek out ways to make care more accessible. Additionally, they should be prepared to answer questions and dispel myths that may hinder patients’ willingness to maintain oral health.

Our multivariable analysis demonstrated that oral-health knowledge was the only independent factor significantly associated with poor oral health status, even after adjusting for potential confounders such as age, sex, diabetes, hypertension, smoking, blood pressure, and lipid levels. This finding indicates that knowledge-related behaviors may outweigh biomedical risk factors in determining patients’ self-reported oral health. While diabetes, smoking, and hypertension are known contributors to oral and cardiovascular disease, their effects did not remain significant in the adjusted model, suggesting that these associations observed in bivariate analyses were confounded. These results are consistent with evidence from other low- and middle-income settings where poor awareness has been shown to mediate the relationship between chronic disease burden and oral health outcomes. From a public health perspective, the data underscore the need to embed oral-health education within cardiovascular care pathways in Odisha, ensuring that patients with CVD and related risk factors are systematically counselled on oral hygiene and preventive practices as part of their long-term disease management.

While this study’s findings are indeed illuminative, it is important to acknowledge its limitations to understand the scope of the implications. One major limitation may be this study’s geographical restriction to the Odisha region, which may affect the generalizability of the results to other demographics and locations. Patients’ oral health perceptions and behaviors could vary significantly according to regional healthcare infrastructure, cultural beliefs, and socioeconomic factors.

However, comparable findings have been reported in other low- and middle-income countries (LMICs), where structural and socioeconomic barriers limit oral healthcare utilization. Similar trends have been observed in sub-Saharan Africa and Southeast Asia, where low awareness of the oral–systemic health connection and infrequent preventive dental visits mirror the challenges seen in our population [28,34,35,40,43,47]. These parallels suggest that the barriers identified in Odisha—such as limited awareness, infrequent dental visits, and socioeconomic constraints—are not unique to India but reflect broader gaps across LMIC health systems. Placing our findings within this global context reinforces the urgent need for integrated policies and culturally adapted oral health education strategies in resource-limited settings.

Another potential limitation is the reliance on self-reported data regarding oral health behaviors, which can introduce bias. Individuals may over-report positive behaviors and under-report negative behaviors. Moreover, the survey design may not capture all the nuanced reasons behind patients’ choices to forgo dental care or neglect oral hygiene, such as fear of dental procedures, personal experiences, or perceived irrelevance. Furthermore, while the survey assessed knowledge via correct/incorrect answers, it did not gauge the depth of understanding or the reasons behind the knowledge gaps. A qualitative assessment could provide richer insights into why patients with CVD or at risk of developing CVD may be less informed or engaged with their oral health.

### 5.1. Mechanistic Considerations Linking Knowledge, Behavior, and CVD Risk

In populations with low oral-health knowledge, behavioral patterns such as infrequent toothbrushing and irregular dental attendance promote dental plaque accumulation and periodontal inflammation, increasing oral microbial burden and dysbiosis. Periodontal pathogens (and their endotoxins/proteases) can gain transient bloodstream access during daily activities, sustaining a low-grade systemic inflammatory state characterized by elevations in CRP, IL-6, and TNF-α and by endothelial dysfunction—changes that contribute to atherogenesis and adverse cardiometabolic profiles (insulin resistance, dyslipidemia, and higher blood pressure). These pathways offer biologically plausible links between inadequate oral-health knowledge (and resultant behaviors) and CVD risk factors. Furthermore, dysbiosis may reduce nitrate-reducing commensals that help maintain nitric-oxide bioavailability, a mechanism implicated in blood pressure regulation. In CVD cohorts, xerostomia associated with commonly used medications can further increase plaque retention; without adequate knowledge and self-care, this iatrogenic risk compounds inflammatory load. Together, these interlocking mechanisms support the observation that individuals with poorer oral-health knowledge exhibit behaviors that heighten microbial burden and systemic inflammation, thereby worsening established CVD risk factors. Our data showing low preventive attendance and suboptimal toothbrushing frequency in this cohort are consistent with these pathways and underscore the need for integrated education within cardiac care (e.g., brushing ≥2×/day, interdental cleaning, and timely periodontal management) [41,42,43,48].

### 5.2. Local Policy Implications

Our findings have direct relevance for Odisha, where cardiovascular and metabolic disease management programs are already expanding. Incorporating oral health education into existing cardiac rehabilitation clinics and discharge counseling for CVD patients could improve patient adherence to preventive practices and reduce long-term disease burden. Given that a significant proportion of patients in our study reported low awareness and poor preventive behaviors, training cardiology nurses and rehabilitation staff to deliver basic oral health advice (e.g., brushing techniques, periodontal disease warning signs, and appropriate referral pathways) could provide immediate benefits at minimal cost. In addition, state-level community health initiatives, such as those led by Accredited Social Health Activists (ASHAs) and Primary Health Centers, could integrate oral health into their routine cardiovascular risk-reduction messaging. Such Odisha-specific interventions would not only address local gaps in knowledge but also serve as a scalable model for other regions of India with similar healthcare challenges.

### 5.3. Strategic Interventions to Optimize Healthcare for Oral Health in CVD Patients [35,43,47,48,49]

- Integrative Care Models: Integrative care is fundamental to improving outcomes for cardiovascular patients. By incorporating dental professionals into cardiovascular teams, healthcare systems can create a more holistic approach to patient care. Regular dialogues between cardiologists, primary care physicians, and dentists can ensure oral health becomes an integral part of cardiovascular management.
- Enhanced Patient Education: Education is key to changing perceptions and behaviors regarding oral health. Developing clear, concise, and culturally sensitive educational materials can help bridge the gap in knowledge among cardiovascular patients. Tailoring these programs for varying literacy levels ensures that all patients can benefit, leading to potentially improved adherence to recommended oral health practices.
- Public Health Campaigns: Widespread public health campaigns that highlight the importance of oral hygiene in relation to overall health are essential. By situating these campaigns within the context of cardiovascular risk, they can effectively target at-risk populations, emphasizing the impact of good oral health on cardiovascular outcomes.
- Cultural Competency Training: Recognizing that cultural norms profoundly influence health behaviors, training healthcare providers to be culturally competent can improve patient-provider communication and foster more inclusive care environments. Understanding different attitudes towards healthcare allows providers to offer more effective, individualized patient education and encourages better health behaviors.
- Expanded Insurance Coverage: Financial barriers often prevent regular dental care. By advocating for more inclusive dental coverage within insurance models, patients may be more inclined to seek preventative measures and treatments for oral health, potentially reducing complications associated with cardiovascular disease.
- Accessible Dental Services: By increasing the number of dental facilities and introducing mobile dental clinics, especially in underserved areas, patients are more likely to receive the care they need. Moreover, providing transport solutions can significantly reduce the inconvenience of attending dental appointments, thus improving adherence to regular dental screenings.
- Screening and Referral Programs: Systemic oral health screenings during cardiovascular clinic visits with an efficient referral system can help detect and treat oral health issues early. Such programs also serve to continually reinforce the importance of oral health to patients.
- Interdisciplinary Training: Ensuring that all healthcare professionals, especially those managing CVD, are educated about the role of oral health in systemic diseases will help integrate oral health evaluations into routine care for cardiovascular patients.
- Community Outreach: Targeted outreach initiatives that focus on educating and providing direct dental care to communities, especially those at higher cardiovascular risk, are essential. Mobile dental units, local health fairs, and partnerships with community organizations can increase accessibility and responsiveness to oral healthcare.

## 6. Conclusions

The survey highlights the urgent need for strategic interventions that target oral health awareness, accessibility, and integration into cardiovascular disease management. Healthcare providers, policymakers, and community leaders must work collaboratively to dismantle the barriers and misconceptions that prevent optimal oral health care for patients with cardiovascular disease and at cardiovascular risk. After adjusting for confounding variables, oral-health knowledge emerged as the key determinant of poor oral health status among patients with cardiovascular disease and risk factors in Odisha. This underscores the need for targeted educational interventions within cardiac rehabilitation and community health programs to improve oral health outcomes in this population. Recognizing and addressing these challenges will be instrumental in the pursuit of holistic patient care and improved overall health outcomes. Future studies should aim to overcome the limitations of this work and perhaps incorporate mixed-method approaches to capture a more comprehensive picture of patients’ health-related decisions and the healthcare system’s strengths and weaknesses in addressing oral health in the context of cardiovascular disease.

## Figures and Tables

**Table 1:** Distributions of patients by age, gender, status of comorbidity, habits, therapy, cardiovascular risks, and descriptive statistics of hemodynamic parameters.

Category	Subcategory	No.	%	Mean ± SD	Median (IQR)	Range (Min.–Max.)
Age distribution	<50	359	69.8	49.0 ± 7.9	47 (43–52)	(40–79)
	50–60	113	22.0			
	>60	42	8.2			
Gender Distribution	Male	363	70.6			
	Female	151	29.4			
Comorbidities and Habits	Diabetic	386	75.1			
	Smoker	423	82.3			
	Hypertension	317	61.7			
	Statin Use	41	8			
	Aspirin Therapy	23	4.5			
Hemodynamic Parameters	SBP (mmHg)			130.3 ± 13.3	131.0 (119.8–140.0)	(96.0–178.0)
	DBP (mmHg)			82.7 ± 9.5	84.0 (77.0–89.0)	(6.0–99.0)
	Total Cholesterol (mg/dL)			174.4 ± 48.3	166.9 (139.8–195.3)	(49.0–402.7)
	HDL (mg/dL)			43.0 ± 7.4	42.1 (39.8–45.2)	(11.9–84.0)
	LDL (mg/dL)			104.5 ± 40.8	98.2 (76.1–126.3)	(24.9–319.0)
	ASCVD Risk (%)			11.7 ± 8.0	9.7 (6.2–15.5)	(0.1–46.0)
Cardio-Vascular Risk (ASCVD score)	Low (<5%)	86	16.7			
	Borderline (5–7.4%)	82	16			
	Intermediate (7.5–19.9%)	274	53.3			
	High (≥20%)	72	14			
Total Cases		514	100			

**Table 2:** Distributions of patients by self-assessment of oral health status.

Self-Assessment	Classification	No.	%
Oral health status	Very good	106	20.6
	Good	331	64.4
	Fair	77	15.0
Self-reported oral health problems	One problem	222	43.2
	Two or more problems	292	56.8
Type of oral health problems	Teeth that do not look right (broken, crooked, discolored)	80	15.6
	Dry mouth	96	18.7
	Sensitivity	161	31.3
	Cavities	68	13.2
	Toothache	26	5.1
	Bleeding/swollen/painful gums	56	10.9
	Loose teeth	0	0
	Other problems	27	5.3
Dental problem affects what you eat	Yes	188	36.6
	No	326	63.4
Importance of oral health compared to overall health	Low importance (0–4)	153	29.8
	Neutral (5)	278	54.1
	Important to extremely important (6–10)	83	16.1

**Table 3:** Oral health behavior.

Practice	Classification	No.	%
Use of partial or full dentures	Yes	126	24.5
	No	388	75.5
How often do you brush your teeth/dentures?	Few times a week	110	21.4
	Less than once per day	95	18.5
	Once a day	295	57.4
	Twice or more times a day	14	2.7
Oral hygiene products used	Fluoride toothpaste	192	37.4
	Dental floss or other aids	174	33.9
	Mouthwash	147	28.6
	Sugar free chewing gum	1	0.2
Seen a dentist in the last 12 months	Yes	46	8.9
	No	468	91.1
When was your last dental visit?	<1 year	42	8.2
	1 year to 2 years	230	44.7
	2 years to 5 years	181	35.2
	5 years	40	7.8
	Do not know	21	4.1
Where do you most often see the dentist?	Private clinic	198	38.5
	Public clinic or hospital (government funded)	226	44.0
	Other	66	12.8
	Do not know	24	4.7
Received information about oral health care since diagnosis	Yes	226	44.0
	No	288	56.0

**Table 4:** Association of OHS with ASCVD risk score.

Oral Health Status	ASCVD Risk Score								Total		*p*
	Low		Border Line		Intermediate		High				
	No.	%	No.	%	No.	%	No.	%	No.	%	
Very good/ Excellent	19	22.1	12	14.6	59	21.5	16	22.2	106	20.6	*p* = 0.561
Good	53	61.6	59	72.0	170	62.0	49	68.1	331	64.4	
Fair	14	16.3	11	13.4	45	16.4	7	9.7	77	15.0	
Total	86	100	82	100	274	100	72	100	514	100	

**Table 5:** Comparison of knowledge score with oral health status.

Oral Health Status	No.	Knowledge Score		ANOVA ‘*p*’ Value
		Mean ± SD	Median(IQR)	
Very good/ Excellent	106	4.9 ± 1.4	5(3–6)	*p* < 0.001
Good	331	5.3 ± 1.1	5(5–6)	
Fair	77	7.5 ± 2.0	9(5–9)	

## Data Availability

The raw data supporting the conclusions of this article will be made available by the authors upon request.

## Supplementary Materials

The following supporting information can be downloaded at https://www.mdpi.com/article/10.3390/dj13090401/s1, File S1. STROBE Checklist for Cross-Sectional Studies.
