# Supplementary material for: Oral Health Status, Behavior, and Knowledge of Patients with Cardiovascular Disease and Associated Risk Factors in Odisha: A Cross-Sectional Survey
Source: Dent J (Basel). 2025 Sep 1;13(9):401. doi: 10.3390/dj13090401 (PMC12468224; doi:10.3390/dj13090401)
Supplement: Supplementary file 1 [file dentistry-13-00401-s001.zip › dentistry-3787875-supplementary.pdf]

File S1. STROBE Checklist for Cross-Sectional Studies

Study title: **Oral health status, behavior and knowledge of patients with cardiovascular disease and associated risk factors in Odisha: a cross-sectional survey**

Item	Recommendation	Reported in manuscript (page/section)
**Title & Abstract**	Indicate the study design in the title/abstract; provide informative abstract	Title page; Abstract
**Introduction - Background/rationale**	Explain background/rationale	Introduction, p. 2–3
**Introduction - Objectives**	State objectives	Aims & objectives, p. 4
**Methods - Study design**	Present study design	Methods, 3.1 Participant Selection
**Methods - Setting**	Describe setting, locations, relevant dates	Methods, 3.1 & 3.2
**Methods - Participants**	Eligibility criteria, sources/methods of selection	Methods, 3.1
**Methods - Variables**	Define all outcomes, exposures, predictors	Methods, 3.3 Questionnaire Tool
**Methods - Data sources/measurement**	Describe data sources/measurement	Methods, 3.2–3.3
**Methods - Bias**	Efforts to address bias	Methods, 3.1–3.3 (informed consent, pilot validation)
**Methods - Study size**	Explain how study size was arrived at	Methods, 3.4 Sample Size
**Methods - Quantitative variables**	Explain handling of quantitative variables	Methods, 3.3–3.5
**Methods - Statistical methods**	Describe all statistical methods	Methods, 3.5 Data Analysis
**Results - Participants**	Report numbers at each stage	Results, 4.1 Demographic Status
**Results - Descriptive data**	Provide participant characteristics, missing data	Results, 4.1–4.3
**Results - Outcome data**	Report outcome data	Results, 4.2–4.3
**Results - Main results**	Unadjusted and adjusted estimates with CI	Results, 4.4–4.6; regression paragraph
**Results - Other analyses**	Report subgroup/sensitivity analyses	Results, 4.6 (sensitivity analysis)
**Discussion - Key results**	Summarize key results	Discussion, p. 19–21
**Discussion - Limitations**	Discuss study limitations	Discussion, p. 20
**Discussion - Interpretation**	Provide interpretation	Discussion, p. 19–21
**Discussion - Generalisability**	Discuss generalisability	Discussion, p. 20

**Other - Funding**	Report funding source	Funding statement
**Other - Ethical approval**	Report ethics approval	Methods, 3.1 (IEC Registration)
**Other - Conflicts of interest**	Disclose conflicts of interest	Conflict of Interest statement
